# Human and farm influences on dairy cows´ responsiveness towards humans – a cross-sectional study

**DOI:** 10.1371/journal.pone.0209817

**Published:** 2018-12-31

**Authors:** Asja Ebinghaus, Silvia Ivemeyer, Ute Knierim

**Affiliations:** Section of Farm Animal Behaviour and Husbandry, Faculty of Organic Agricultural Sciences, University of Kassel, Witzenhausen, Germany; University of Illinois, UNITED STATES

## Abstract

The human-animal relationship can have a major impact on cow welfare and stockpersons´ work safety and quality. This cross-sectional study investigated possible effects of stockperson and farm related factors on cows´ behaviour towards humans in different test situations on a range of farm types including farms with automatic milking system. On 32 dairy farms, cows´ avoidance distances (AD), tolerance to tactile interactions (TTI), behavioural reactions during and after release from restraint (RB) and expressive behaviour by means of qualitative behaviour assessment (QBA) were recorded. Multiple regressions were calculated to analyse associations between the cows´ responses towards humans and factors of stockpersons´ attitudes, factors of human-animal contact and further herd and management characteristics. Positive attitudes towards cows were significantly associated with calmer cows in all test situations. Furthermore, different herd and management factors were related to individual variables of cows´ behaviour towards humans: for instance, the percentage of dehorned cows in the herd was associated with more fearful cows; the frequency of human-animal contact, manual feed provision, or selection for docility were associated with calmer cows. Explained variances were highest for the outcome variables AD and QBA. Directly or indirectly all factors remaining in the final models may be related to the amount and quality of human-animal contacts. Thus, the results suggest that on a broad range of different farm types a positive attitude and frequent human-animal contact can contribute to calmer cows in different interactions with humans in the barn.

## Introduction

The importance of the human-animal relationship (HAR) in the context of farm animal welfare and production as well as stockpersons´ work safety has been increasingly recognized over the last decades (reviewed by [1–4]). In dairy farming, the quality of the HAR can influence milk ejection and milk yield (e.g. [5–8]), success at first insemination [9], aspects of udder health [10] or the incidence of lameness [11]. Moreover, positive handling and less fearful cows may improve work safety and quality for the stockperson, e.g. due to less stepping and kicking behaviour during the milking routine [12,13], during veterinary treatment [14], or when moving the cows to claw trimming [15].

Waiblinger et al. [3] defined the HAR as the degree of relatedness or distance between human and animal. The HAR is based on both, the experiences of the human and the animal, and reflects the mutual perception and evaluation. Accordingly, the stockpersons´ attitudes and behaviour towards the animals are considered as key factors determining the quality of daily human-animal interactions and the animals´ confidence in or fear of humans [1,16]. Most intensively investigated are associations between stockpersons’ negative or positive interactions with the cows in the milking parlour and cows´ behaviour towards humans (e.g. [7,9]), while various studies have shown that the stockpersons´ behaviour is affected by their attitudes and personality traits [5,7,9,17,18]. Positive and empathic attitudes towards cows, for instance, were positively correlated with the number or percentage of observed positive interactions during milking, such as talking patiently, touching or stroking the cows [7,9,19]. Stockpersons´ attitudes are the basis for their behaviour and decisions concerning immediate handling of their animals and can be expected to be indirect predictors for the animals´ behaviour towards humans. Moreover, attitudes have been shown to more broadly affect farmers´ animal welfare related decisions in terms of management and housing [20,21], which again might be directly or indirectly related to cows´ behaviour towards humans.

Besides the quality of stockpersons´ interactions with the cows also the intensity and continuity of human-animal contact were found to be associated with cows´ behaviour towards humans: des Roches et al. [22] found a higher percentage of cows that allowed touching in the avoidance distance test on farms with a closer ratio of cows per stockperson [22]. In Swiss dairy herds, increased daily contact time and the stockpersons´ ability to identify all individual cows were related to less cows with high avoidance distances [19]. In investigations by Waiblinger et al. [23] a combined variable including frequency of brushing, identification of cows, gentle handling, number of milkers and frequency of personnel changes was related to less avoidance behaviour of cows towards humans.

Previous on-farm studies regarding influencing factors on the cows´ behaviour towards humans have also found relations between herd and management characteristics and the HAR. For instance, Waiblinger and Menke [24] found that the herd size correlated negatively with the percentage of cows that allowed touching by an approaching human, which could be attributed to reduced intensity of contact between human and cow in larger herds. Waiblinger et al. [23] showed that on farms with milking parlour also feeding management practices including close handling of animals (e.g. confining the cows individually in the feeding gate) and by tendency a lower percentage of dehorned cows in the herd were associated with less avoidance behaviour of cows towards humans.

However, the reported associations may depend on actual on-farm conditions. Considering the broad range of possible farm types with their widely varying settings and, additionally, the necessarily limited sample sizes of on-farm studies, it appears that more investigations of this type are needed. For instance, only one recent French study also included farms with automatic milking systems (AMS) [22], which may profoundly alter frequency and type of interactions between humans and cows.

In addition, previous cross-sectional studies exclusively used the established HAR-measure ‘avoidance distance’ (AD), whose validity has been confirmed in various on-farm and experimental studies (reviewed in [3]). A first analysis regarding criterion validity of further HAR-measures using the present data set (including an additional farm for which no data on stockpersons´ attitudes could be recorded) suggests that other HAR-measures might reflect additional or partly different aspects of the HAR [25]. In contrast to AD, which involves mere approach of a human towards the animal, these measures test the cow’s response in standardised handling situations.

The aim of the present cross-sectional study was therefore to explore patterns of influences on the cows´ responsiveness towards humans in different test situations, taking into account stockpersons´ attitudes, human-animal contacts as well as herd and management characteristics of different farm types including farms using AMS.

## Farms, animals, material and methods

### Farms and animals

In total, 32 dairy farms (24 organic and 8 conventional farms) located in Middle and Northern Germany were assessed in two winter periods from December 2014 to March 2016. Assessments were done during winter to enable observations of cow behaviour in the barn also on farms providing pasture access during summer. All farms had loose housing systems, took part in regular milk recording schemes and had > 50% Holstein Friesian cows in their herds. Herd sizes ranged from 29 to 530 (mean ± sd = 102.7 ± 106.8) and the average daily milk yield ranged from 17.9 to 36.1 kg (mean ± sd = 23.8 ± 4.2). Summer pasture was offered for all cows on 23 farms, on four farms only for dry cows, and five farms had zero grazing. While 11 organic farms kept horned cows, the other 13 organic and eight conventional farms kept dehorned and partly genetically hornless cows. Nine farms used automatic milking systems (AMS), the other 23 farms had fishbone or tandem milking parlours. 23 farms were family-operated, while the others were farm communities or agricultural cooperatives. Since the cows had to be fixed in the feeding gate for the application of two of the behavioural tests, all farms were at least partly equipped with self-locking feeding gates.

### Data collection

The cows´ behaviour towards humans was assessed by direct observations in four behavioural tests. Potential influencing factors were recorded by a survey of stockpersons´ attitudes towards cows via questionnaire, and by documenting factors of human-animal contact, herd and management characteristics. Stockpersons´ interactions towards cows were recorded during milking on farms with parlour systems. However, due to the reduced sample size (n = 23), these data were not included in the present data analyses.

Each farm was visited once and all data recordings took place on one or at maximum two consecutive days. Depending on herd size and milking routine, the duration of farm visits ranged from 8–12 h (one day) to 16 h (two days; including observations in the milking parlour, data not reported here).

The study did not involve endangered or protected species, aversive animal handling or invasive procedures. Observations of animal behaviour were conducted under unchanged farm conditions. The participation of farms in the study was voluntary, and the farmers were informed about the purpose and methods of the study by written project information in advance. They were assured that all information would be treated anonymously, and that they could withdraw from the study at any time. For the use of herd data, written farmers´ consents were obtained.

#### Cows´ behaviour towards humans

The following measures were applied according to Ebinghaus et al. [25,26] at individual animal level: avoidance distances towards an unfamiliar person at the feeding place (AD), tolerance to tactile interaction (TTI), behaviour during release from restraint (RB), and the cows´ body language in a standardised human-animal interaction, here during the TTI and RB test, by qualitative behaviour assessment (QBA).

Depending on herd size, AD, TTI, and RB were recorded in 26 to 100% of dairy cows per farm, including cows of all parities (sample size determined according to Welfare Quality [27]). Measures were not applied in pregnant heifers, and in dry cows only when kept in the same group with lactating cows. Since the application of QBA requires a high level of concentration from the experimenter, this measure was conducted on a sample of 11 to 30 cows per farm. Data collection was carried out by altogether seven trained experimenters (six females, one male; wearing similar green overalls and gumboots; varying in body heights, from 165–183 cm). Five of them performed and assessed AD, TTI, and RB alternatingly within and between farm visits; two experimenters additionally performed QBA. The experimenters were all having a minimum experience in working with cows or in behavioural observation, but on different levels. After joint training, prior to data collection, inter-observer reliability regarding estimation of the AD, scoring of the cows’ responses in the TTI and RB test, and applying QBA was tested by direct observations at the research farm of the University of Kassel, Germany. For this purpose, the experimenters alternatingly performed the tests, while the other experimenters located on the feed bunk assessed the cows´ responses from a distance of about 3 m. For reliability testing of TTI and RB video material was used, additionally. Acceptable agreements were achieved for all experimenters and measures (r_s_ = 0.71–0.94, PABAK = 0.56–0.91).

Behavioural observations always started after feeding in the morning, when the cows were restraint in self-locking feeding gates. On farms with conventional milking parlour, feeding took place always after milking and on farms with AMS within the time range from 6:30 to 9:30 a.m. Individual cows were then assessed by means of the behavioural measures. All four tests were always applied in a row and in the same order on each cow: at first, one experimenter, located on the feed bunk, measured the AD. Subsequently, another experimenter located on the barn side of the feeding gate, and blind to the assessment of AD, carried out the TTI and RB tests as well as the QBA. For the TTI test the experimenter looked at the fixed test cow from behind, left and right hand side for about 30 s, then approached the cow from one side, stroked three times along the back and down the flank. The behavioural reaction was rated on a 5-point scale; 1: cow stays calm, 2: cow lowers hindquarters at least over the duration of two strokes, or steps at maximum twice, 3: cow steps three to five times, 4: cow steps more than five times or kicks at least once, 5: cow reacts violently, touching not or barely possible. RB was then assessed during and after opening the feeding gate by the experimenter located at the barn side, and also rated on a 5-point scale; 1: cow stays calm, leaves the feeding place hesitantly, 2: cow leaves in intermediate speed, 3: cow leaves walking fast, 4: cow leaves running or jumping, 5: cow reacts violently, opening of feeding gate barely possible. AD, TTI, and RB were recorded using digital record sheets on Sony Xperia Z2 tablet computers. Afterwards, the experimenter located on the barn side performed the QBA using a fixed list of 20 descriptors, which had been developed for this purpose [26] and was based on Wemelsfelder et al. [28]. On visual analogue scales the degree of expression of each descriptor was marked using the software QBA App 1.0.7 (www.egenes.co.uk/qba). The multivariate QBA data of all assessed cows were reduced to at maximum two dimensions by means of principal component analysis (PCA, eigenvalue > 1, correlation matrix, without rotation, SPSS 24). The PCA explained 67.5% of variance on the first principal component (PC1), which was characterised by descriptors relating to relaxation/attraction/trust on the negative and descriptors relating to fear/distress/aversion on the positive end.

For data analysis, medians at farm level were calculated for AD (ADmedian) and QBA scores of PC1 (QBAmedian). With regard to AD also the percentages of cows per farm that could be touched (ADtouch) and that avoided the approaching human at a distance ≥ 100 cm (AD100) were calculated. Regarding TTI and RB, the percentages of fearful cows scoring > 2 were used (TTIhigh and Rbhigh, Table 1). The term “fearful” is used in this paper as a proxy for the range of different aversive behavioural responses to the HAR-tests.

**Table 1:** Descriptive data on cows´ responsiveness towards humans assessed by different measures in the barn; summarised at herd level.

Measures	n	mean ± sd	median	min—max
ADmedian (cm)	32	19.4 ± 16.8	15.0	0.0–70.0
ADtouch (%)	32	26.9 ± 18.2	22.8	0.0–71.0
AD100 (%)	32	9.0 ± 9.4	5.3	0.0–40.1
QBAmedian*	32	-0.041 ± 0.644	0.101	-1.254–1.089
TTIhigh (%)	32	30.9 ± 12.2	30.2	0.0–53.9
RBhigh (%)	32	26.0 ± 12.6	26.9	3.5–54.1

ADmedian = median of avoidance distances, ADtouch = percentage of cows that allowed touching in the AD test; AD100 = percentage of cows that avoided the approaching human at ≥ 100 cm, QBAmedian = median of qualitative behaviour assessment PC1-scores, TTIhigh = percentage of more fearful reacting cows in the tolerance to tactile interaction test (score > 2), RBhigh = percentage of more fearful reacting cows in the release behaviour test (score > 2)

* negative values relate to relaxation/attraction/trust, positive values relate to fear/distress/aversion

#### Stockpersons´ attitudes towards cows

A questionnaire containing 51 questions on stockpersons´ attitudes regarding (1) handling when moving the cows, (2) handling during milking, (3) importance of certain general handling practises, and (4) on the personal perception of different human-cow contacts was used ([29], modified after Waiblinger et al. [7]). All farm staff involved in work with cows was asked to participate in the survey. The questionnaire was designed to be filled in by the participant independently within about 20 min. Beforehand the questionnaire´s design was shortly explained by the researchers. The answers were always given on 7-point Likert scales. Finally, data on age, work experiences and professional training were asked. Participants filled in the questionnaire during or shortly after the farm visit.

Answers (items) were grouped within the four questionnaire topics using a principal component analysis (PCA, maximum 2 principal components, eigenvalue > 1.0, varimax rotation, SPSS 24). Only items loading ≥ 0.3 on one principal component were integrated in the further analysis. When items were assigned to two components, loadings had to be ≥ 0.6 on the first and ≤ 0.4 on the second component, and items were only selected for the first component [7]. Items that did not fulfil these requirements were excluded from further analysis. According to the resulting eight principal components, eight new factors were assigned (two factors in each questionnaire topic, Table 2). For each new factor, Likert-scale scores of the assigned items were averaged.

**Table 2:** Factors of stockpersons´ attitudes towards cows created from questionnaires by means of PCA.

Factors	included items	no. of items
moveFORCE	agreement on forcing, punishing behaviour when moving cows (e.g. use of stick, cows must not pause)	10
moveMILD	agreement on patience when moving the cows (use of voice and hand)	3
milkFORCE	agreement on punishment when a cow kicks during milking (e.g. shouting)	4
milkPOS	agreement on talking calmly when a cow kicks during milking	4
importancePOS	agreement on importance of positive human-animal contact (e.g. speaking to cows in the barn, stroking)	6
importanceCONTROL	agreement on importance of contact to monitor cows (e.g. controls in the barn, observing cows)	5
contactVOL	voluntary and tactile contact to cows is perceived as pleasant (e.g. stroking, tactile contact during milking)	5
contactNEED	necessary contact to cows is perceived as pleasant (treatment of ill cows, assistance at calving)	2

If more than one person per farm had answered the questionnaire, weighted averages were calculated: irregularly involved family members were weighted with the factor 0.5, non-qualified and non-permanent staff with the factor 0.7; farm managers, permanent staff, and regularly involved family members with the factor 1.0.

#### Herd, housing and management characteristics

Altogether 21 herd, housing and management factors (including factors regarding the quantity and type of human-animal contacts) potentially related to the HAR were considered in accordance with previous studies [7,10,23]. Data on human-animal contacts, herd characteristics and management were collected via questionnaire-guided interviews with the farm or dairy herd manager; data on housing were collected during the farm visits using standardised recording sheets.

Regarding **human-animal contacts**, the number of cows per stockperson, contact time of all farm staff ‘on foot’ per cow (i.e. milking and work in the barn e.g. cubicle care, manual provision of roughage, moving animals; excluding time near animals on machines), contact time of all farm staff ‘on foot’ with calves (i.e. feeding and work in the barn; excluding time near animals on machines), and the manner of roughage and concentrate provision (manually/by machine) were recorded. Furthermore, active habituation of heifers to humans, frequencies of controls in the barn, claw trimming and staff changes, and voluntary contacts to lactating cows, dry cows and to calves were recorded. Voluntary contacts were quantified according to the weekly frequencies of different interactions beyond routine work (observing, brushing, speaking to animals, udder control in cows; observing, touching, speaking in calves). Each interaction was multiplied by a factor, depending on the frequency stated by the stockpersons (categories: twice daily, daily, every 2–3 days, once a week, less than once a week (only in calves), never), ranging from four (cows) to five (calves), if carried out twice daily, to zero, if never carried out. Thus, each farm could reach up to 16 points for contacts with cows and 15 points for contacts with calves. The measure was expressed as the percentage of actual points in relation to the maximal points. During the farm visits the stockpersons’ ability to identify individual cows was recorded (yes/no).

Data on **herd, housing and further management characteristics** included the herd size and percentage of dehorned cows in the herd (excluding genetically hornless cows), selection for docility (yes/no), and the milking system. Finally, the situation during feeding and the type of feeding gate (palisade/diagonal), which might affect the cows´ behaviour during release from restraint and thus influence the rating regarding RB and QBA, were recorded.

### Statistical data analyses

Multivariable data analyses were carried out for six outcome variables (ADmedian, ADtouch, AD100, TTIhigh, RBhigh, QBAmedian). Graphical tests via normal quantile-quantile plots [30] showed that metric data of cows´ behaviour as well as metric data of environmental factors were partly non-normally distributed. Thus, univariable pre-selection of variables for subsequent multivariable analyses was carried out using non-parametric analyses: Spearman rank correlation (r_s_) in the case of metric variables, Kruskal-Wallis tests for categorical, and Mann-Whitney-U tests for dichotomous variables. Only variables with p ≤ 0.1 were selected for multivariable analyses. Metric and ordinal variables strongly correlating with other variables (r_s_ > 0.7, S1 Table) and nominal variables with similar contents were not included in the same model to avoid multicollinearity. In those cases, the variable with the strongest association to the outcome variable was chosen. In the results of univariable pre-selection, regardless of parametric or non-parametric analyses, means are presented (Table 3).

**Table 3:** Univariable pre-selection of potentially influencing factors concerning HAR; based on Spearman rank correlations (r_s_) or group mean differences (Kruskal-Wallis / Mann-Whitney U test); selected factors for multivariable regression are marked in bold (n = 32).

	descriptive data			Spearman rank correlations: r_s_ (p-value)					
	median	mean ± sd	min—max	ADmedian	ADtouch	AD100	TTIhigh	RBhigh	QBAmedian
herd size (number of cows)	79.0	102.69 ± 106.82	29–530	**0.52 (0.003)**	**-0.51 (0.003)**	**0.46 (0.009)**	**0.32 (0.075)**	**0.34 (0.054)**	**0.53 (0.002)**
% dehorned cows (excl. genetically hornless cows)	82.5	56.47 ± 45.40	0.00–100.00	**0.43 (0.013)**	**-0.39 (0.026)**	**0.51 (0.003)**	**0.51 (0.003)**	**0.47 (0.007)**	**0.57 (0.001)**
number of stockpersons (incl. managers, employees, trainees)	3.8	4.81 ± 4.35	2.00–25.00	0.09 (0.607)	-0.13 (0.507)	0.13 (0.490)	-0.18 (0.316)	0.01 (0.939)	0.01 (0.963)
number of cows per stockperson	22.7	24.06 ± 14.35	4.40–63.57	**0.40 (0.025)**	**-0.38 (0.031)**	**0.32 (0.071)**	**0.31 (0.090)**	0.23 (0.201)	**0.44 (0.012)**
contact time per cow (min/d), ‘on foot’^1^, including milking	5.3	7.60 ± 7.24	0.59–32.58	**-0.47 (0.007)**	**0.47 (0.006)**	**-0.39 (0.026)**	-0.27 (0.141)	**-0.36 (0.044)**	**-0.56 (0.001)**
total contact time calves(min/d), ‘on foot’^1^, including feeding	82.5	105.16 ± 97.70	15.00–510.00	-0.13 (0.496)	0.13 (0.466)	0.06 (0745)	-0.14 (0.463)	-0.14 (0.454)	-021 ((0.245)
voluntary contact to cows (%)^2^	50.0	50.39 ± 14.19	12.50–81.25	-0.07 (0.719)	0.09 (0.624)	0.08 (0.675)	**-0.31 (0.082)**	-0.08 (0.661)	-0.12 (0.520)
voluntary contact to dry cows (%)^2^	50.0	47.46 ± 17.81	18.75–81.25	**-0.43 (0.013)**	**0.39 (0.028)**	-0.25 (0.177)	**-0.45 (0.011)**	**-0.43 (0.013)**	**-0.49 (0.004)**
voluntary contact to calves (%)^2^	76.7	74.38 ± 19.46	33.33–100.00	**-0.35 (0.051)**	0.20 (0.270)	**-0.30 (0.096)**	**-0.34 (0.061)**	-0.29 (0.105)	**-0.47 (0.007)**
stockpersons´ attitude^3^									
moveFORCE	3.5	3.5 ± 0.7	1.9–5.1	0.02 (0.901)	0.06 (0.742)	0.15 (0.420)	-0.10 (0.577)	0.20 (0.277)	-0.02 (0.903)
moveMILD	5.5	5.4 ± 0.7	3.7–7.0	0.29 (0.111)	-0.19 (0.310)	0.11 (0.569)	-0.17 (0.348)	-0.03 (0.862)	0.13 (0.486)
milkFORCE^4^	2.7	2.5 ± 0.7	1.0–3.9	0.05 (0.810)	-0.01 (0.974)	0.24 (0.196)	-0.04 (0.841)	0.06 (0.753)	-0.08 (0.682)
milkPOS^4^	5.9	5.7 ± 0.6	4.0–6.6	-0.21 (0.267)	0.16 (0.382)	-0.17 (0.362)	-0.44 (0.014)	-0.32 (0.083)	-0.33 (0.073)
importancePOS	5.6	5.5 ± 0.8	4.0–6.9	**-0.38 (0.031)**	**0.33 (0.067)**	**-0.44 (0.012)**	**-0.40 (0.024)**	**-0.49 (0.005)**	**-0.54 (0.001)**
importanceCONTROL	5.7	5.6 ± 0.8	3.0–6.8	**-0.32 (0.072)**	0.17 (0.348)	**-0.30 (0.098)**	**-0.31 (0.089)**	**-0.51 (0.003)**	-0.29 (0.105)
contactVOL	5.9	6.0 ± 0.5	4.7–6.8	**-0.37 (0.036)**	0.24 (0.189)	**-0.60 (0.000)**	**-0.39 (0.029)**	**-0.51 (0.003)**	**-0.38 (0.031)**
contactNEED	5.2	5.2 ± 0.8	3.5–7.0	-0.22 (0.219)	0.14 (0.455)	-0.23 (0.206)	-0.26 (0.149)	**-0.34 (0.061)**	**-0.33 (0.065)**
		**number**	**percentage**	**Kruskal-Wallis / Mann-Whitney U: mean (p-value)**					
frequency of claw trimming				**(0.097)**	(0.286)	**(0.052)**	(0.626)	(0.303)	(0.343)
if needed		6	18.8	7.92	33.84	1.93	25.67	18.02	-0.278
once a year		6	18.8	12.08	34.40	7.56	31.21	29.40	-0.213
twice a year		14	43.8	26.61	23.30	10.79	31.57	26.16	-0.016
more than twice a year		6	18.8	21.25	20.89	13.07	34.36	29.94	0.309
selection for docility				(0.129)	**(0.096)**	(0.115)	(0.750)	(0.305)	(0.600)
no		25	78.1	21.70	24.53	10.12	31.10	27.15	-0.021
yes		7	21.9	11.07	35.37	4.79	30.30	21.67	-0.112
cow:feeding place ratio^5^ [32–34]				(0.239)	(0.354)	(0.183)	(0.386)	(0.139)	(0.453)
suboptimal		10	31.3	27.50	22.18	13.41	35.51	29.69	0.157
appropriate		10	31.3	18.50	23.97	8.62	29.83	29.15	-0.047
generous		12	37.5	13.33	33.29	5.51	28.01	20.17	-0.201
feeding gate type				(0.124)	(0.167)	**(0.010)**	**(0.001)**	**(0.023)**	**(0.018)**
diagonal self-locking		22	68.8	21.14	23.44	10.24	35.62	29.34	0.142
palisade self-locking		10	31.3	15.50	34.53	6.11	20.59	18.49	-0.444
routine fixation for feeding				**(0.069)**	(0.138)	**(0.009)**	**(0.065)**	(0.138)	**(0.004)**
no		18	56.3	23.89	22.93	12.38	34.39	28.86	0.260
yes		14	43.8	13.57	32.01	4.55	26.47	22.22	-0.428
roughage provision				**(0.005)**	**(0.008)**	**(0.007)**	**(0.013)**	**(0.016)**	**(0.003)**
only by machine		17	53.1	26.18	18.44	12.62	35.07	31.02	0.272
(also) manually		15	46.9	11.67	36.49	4.80	26.22	20.21	-0.395
concentrate provision (barn)				(0.163)	(0.143)	**(0.014)**	**(0.053)**	(0.208)	**(0.015)**
only by machine/station		22	68.8	22.05	23.70	11.09	33.99	28.04	0.168
(also) manually		10	31.3	13.50	33.96	4.25	24.18	21.37	-0.500
milking system				(0.121)	(0.141)	(0.761)	(0.555)	(0.521)	**(0.013)**
AMS		9	28.1	28.06	19.89	12.82	29.86	28.75	0.338
fishbone		19	59.4	18.03	27.72	7.88	31.80	25.86	-0.061
tandem		4	12.5	6.25	38.81	5.36	29.12	20.08	-0.797
staff changes				(0.140)	**(0.086)**	(0.302)	**(0.022)**	(0.253)	**(0.030)**
no change within last years		11	34.4	25.46	18.06	10.57	38.66	31.04	0.352
< once a year		9	28.1	18.06	26.16	8.15	28.45	25.57	-0.103
> once a year		12	37.5	14.79	35.57	8.07	25.68	21.58	-0.355
active habituation of heifers to humans				(0.130)	(0.119)	(0.585)	(0.726)	(0.414)	(0.119)
no		20	62.5	22.88	22.45	9.90	31.06	27.05	0.095
yes		12	37.5	13.54	34.34	7.38	30.69	24.12	-0.267
identification of cows				**(0.026)**	**(0.011)**	(0.645)	(0.954)	(0.687)	(0.199)
no		13	40.6	27.12	16.18	10.48	30.57	27.26	0.144
yes		19	59.4	14.08	34.24	7.91	31.17	25.06	-0.168
frequency of control rounds in the barn				(0.498)	(0.268)	(0.773)	(0.109)	(0.440)	(0.775)
maximum every couple of days		3	9.4	16.67	25.42	6.96	40.69	32.79	0.072
daily		7	21.9	24.29	16.90	10.68	33.95	27.68	0.058
several times daily		22	68.8	18.18	30.29	8.68	28.63	24.47	-0.088
no. of selected factors				13	10	13	14	10	15

^1^ excluding time near the animals, but on machines

^2^ based on weekly frequencies of different human-animal interactions beyond routine work listed in the questionnaire and named by the interviewed farmers (observing animals, speaking to animals, brushing, and udder control in cows; observing, speaking to animals and touching in calves)

^3^ abbreviations see Table 2, score 1 = no agreement, score 7 = full agreement

^4^ n = 31, milkPOS was excluded from multivariable modelling due to a strong correlation (r_s_ = 0.71, p < 0.001) with importancePOS

^5^ based on recommendations: suboptimal: < 1:1.0 in milking parlour systems, < 1:0.8 in AMS systems; appropriate: 1:1.0 in milking parlour systems, 1:0.8–1:1.0 in AMS systems; generous: > 1:1.0

Multivariable linear regression models were fitted with bidirectional stepwise selection of variables according to the Akaike information criterion (AIC) [31]. Model diagnostics were done by graphical evaluation of the residual distribution and the residual by predicted values plot. Absence of multicollinearity was checked by the variance inflation factor (VIF, < 4.0) and absence of influential data points was checked by Cooks´ distance (< 1.0). The level of significance used was α = 0.05, and the results are referred to as tendencies in case of 0.05 < α ≤ 0.1.

Analyses were performed using the software R Studio 1.0.136 for Mac OS X (R Core Team 2016). The package ‘stats’ was used for modelling by AIC, the package ‘lm.beta’ was used to add standardised regression coefficients and the package ‘car’ for multicollinearity tests.

## Results

### Descriptive statistics

Regarding cow behaviour and stockpersons´ attitudes, the investigated farms varied markedly (S2 and S3 Tables). Descriptive data on cow behaviour are shown in Table 1. Factors of stockpersons´ attitudes created from the questionnaires by means of PCA are presented in Table 2. The characterisation of the investigated farms with regard to herd, management and housing factors that may influence the cows´ responsiveness towards humans is shown in Table 3.

The questionnaire regarding attitudes towards the cows was answered by a total number of 97 stockpersons on 32 farms (1–6 persons per farm, 62% male, 38% female) regularly involved in handling the cows. They ranged from 16 to 77 years of age (36.0 years ± 14.9 years), and their work experience with cows ranged from 3 months to 60 years (19.5 years ± 16.2 years). 33% of participants were farm managers, 26% permanent staff, 19% regularly and 3% irregularly involved family members, and 20% non-permanent staff (mainly apprentices or interns).

### Univariable pre-selection of factors

Spearman rank correlations (r_s_) or group mean differences (Kruskal-Wallis / Mann-Whitney U test) of all potentially influencing factors on the six outcome variables are presented in Table 3.

### Multivariable analyses

Significant final models were obtained for all outcome variables (Table 4). The models comprised between two and four factors, and the variances explained ranged between an adjusted R^2^ = 0.322 for the model for TTIhigh and an adjusted R^2^ = 0.697 for the model for QBAmedian.

**Table 4:** Final multivariable linear regression models regarding ADmedian, ADtouch, AD100, TTIhigh, RBhigh, and QBAmedian (n = 32).

ADmedian	Estimate^5^	Standardised^6^	SE	t^7^	p
(Intercept)	110.971	0.000	31.250	3.551	0.001
attitude: contactVOL^1^	-15.369	-0.420	5.065	-3.034	0.005
identification of individual cows	-11.899	-0.354	4.357	-2.731	0.011
% dehorned cows	0.123	0.051	0.051	2.403	0.023
adjusted R^2^ = 0.484, F = 10.680, p < 0.001, VIF = 1.007–1.150					
**ADtouch**					
(Intercept)	-10.886	0.000	7.605	-1.431	0.163
identification of individual cows	13.235	0.364	4.846	2.731	0.011
selection for docility	16.503	0.382	6.022	2.740	0.011
voluntary contact dry cows (%)	0.555	0.544	0.143	3.892	0.001
adjusted R^2^ = 0.484, F = 10.680, p < 0.001, VIF = 1.063–1.171					
**AD100**					
(Intercept)	47.461	0.000	18.296	2.594	0.015
attitude: contactVOL^1^	-7.517	-0.368	2.915	-2.578	0.016
herd size	0.030	0.337	0.013	2.363	0.025
% dehorned cows	0.056	0.273	0.028	2.002	0.055
adjusted R^2^ = 0.522, F = 12.280, p < 0.001, VIF = 1.207–1.318					
**TTIhigh**					
(Intercept)	51.359	0.000	14.067	3.651	0.001
% dehorned cows	0.117	0.437	0.041	2.845	0.008
attitude: importancePOS^2^	-4.896	-0.316	2.379	-2.058	0.049
adjusted R^2^ = 0.322, F = 8.370, p = 0.001, VIF = 1.079					
**RBhigh**					
(Intercept)	86.747	0.000	15.134	5.732	<0.001
attitude: importancePOS^2^	-6.102	-0.381	2.324	-2.626	0.014
palisade feeding gate (reference: diagonal)	-6.277	-0.235	3.868	-1.623	0.116
(also) manual roughage provision	-7.550	-0.304	3.755	-2.011	0.055
attitude: importanceCONTROL^3^	-3.859	-0.247	2.265	-1.704	0.100
adjusted R^2^ = 0.443, F = 7.163, p < 0.001, VIF = 1.163–1.271					
**QBAmedian**^**4**^					
(Intercept)	2.731	0.000	0.501	5.453	<0.001
attitude: importancePOS^2^	-0.435	-0.530	0.097	-4.481	<0.001
milking system (reference: AMS)					
fishbone parlour	0.160	0.124	0.168	0.950	0.351
tandem parlour	-0.752	-0.392	0.222	-3.396	0.002
staff changes (reference: no change within last years)					
< once a year	-0.290	-0.205	0.168	-1.722	0.097
> once a year	-0.492	-0.376	0.157	-3.130	0.004
(also) manual concentrate provision	-0.334	-0.244	0.157	-2.129	0.043
adj. R^2^ = 0.697, F = 12.900, p < 0.001, VIF = 1.187–1.434					

^1^ voluntary and tactile contact to cows is perceived as pleasant

^2^ agreement on the importance of positive human-animal contact

^3^ agreement on importance of contact to monitor cows

^4^negative values relate to relaxation/attraction/trust, positive values relate to fear/distress/aversion

^5^ estimated regression coefficient

^6^ standardised regression coefficient

^7^ value of the t-statistic used to calculate p-values

To each HAR-measure (except for ADtouch) a factor describing the degree of positive attitudes towards interacting with cows (importancePOS) or the degree of positive affective attitudes (contactVOL) showed significant associations (Table 4). The models for ADmedian, ADtouch, RBhigh and QBAmedian also contained one to two factors describing or being the result of the quantity or quality of human-animal contacts (voluntary contact to dry cows, manual roughage or concentrate provision, identification of individual cows). Housing or management factors associated with HAR measures were the percentage of dehorned cows (ADmedian, AD100, TTIhigh), selection for docility (ADtouch), herd size (AD100) and milking system (QBAmedian).

## Discussion

This explorative cross-sectional study on 32 dairy farms aimed at depicting patterns of influences on the cows´ behaviour towards humans using different behavioural measures. Potentially influencing factors of stockpersons´ attitudes, quantity and quality of human-animal contacts, as well as herd and management characteristics were taken into account.

### Evaluation of the sample of farms

The investigated sample of farms covered a range of different farm conditions regarding herd and management characteristics. The investigation included farms with small to large herd sizes, farms with milking parlour and automatic milking systems, organic and conventional farms as well as farms with dehorned or horned cows. Descriptive analyses showed that the farms also differed markedly in terms of human-animal contact and in terms of the cows´ behavioural reactions in different test situations. The avoidance distances (AD) found on the investigated farms were on a similar level and range compared to reports from 30 Austrian and 46 Swiss dairy herds (ADmedian: 19 cm compared to 17 cm [7] and 26 cm [10]; 23% (median) of cows that allowed touching compared to 19% [7] and 28% [10]). It should be noted, however, that in the cited studies AD was measured in the barn, whereas we tested cows at the feeding place. AD can be expected to be slightly higher in the barn than at the feeding gate [26,35]. On the other hand, in 118 French dairy herds only 9% of cows could be touched at the feeding place [22] compared to 27% in the present investigation. Thus, level and range of cows´ behaviour appear to differ depending on the structure of the farm sample, e.g. regarding the share of different herd sizes or milking systems. This should be taken into account when comparing results between studies.

The surveyed stockpersons in the present study agreed more strongly with positive than with negative statements. Agreement on punishment when a cow kicks during milking (milkFORCE), for instance, ranged from 1.0 to 3.9 (on a scale from 1.0 to 7.0), whereas agreement on talking calmly when a cow kicks during milking (milkPOS) ranged from 4.0 to 6.6 (on a scale from 1.0 to 7.0). This pattern is in line with previous studies, using a similar questionnaire and the same method of data merging [7,19].

### Evaluation of the measures

In the present investigation cows´ behaviour towards humans was assessed by the distance measure AD and by the handling measures TTI, RB, and QBA. AD is an established HAR measure, being widely used and recommended [3,27]. In previous studies, AD had been tested for different aspects of reliability, including inter-observer and inter-experimenter reliability, i.e. consistency of cows´ responses towards different experimenters (e.g. [26,35,36]). AD had been validated on different levels, both experimentally and on-farm, and shown to be associated with the quality and quantity of human-animal contact (e.g. [9,14,23]). The handling measures TTI, RB, and QBA had previously only been tested for inter-observer, intra-observer and test-retest reliability and for criterion validity [25,26]: significant inter-test-correlations with AD measures ranged from a moderate r_s_ = -0.57 (between TTIhigh and AD100) to a high r_s_ = 0.75 (between RBhigh and ADmedian) [25]. By assessing the cows´ responsiveness in test situations involving more intense human-animal interactions, these measures appeared to reflect partly different or additional aspects of the HAR [25,26]. However, looking at the present results, no clear systematic differences between the established distance measure AD and the handling measures TTI, RB, and QBA become apparent. Nevertheless, the number of factors and the explanatory value of the final models varied: the model regarding TTIhigh comprised only two factors and had the lowest adj. R^2^ (0.322). Obviously, further factors not considered in the present study, or possibly even inconsistencies of cows´ responses towards different experimenters, contributed to the variation in TTI-responses between farms. RBhigh was not only related to attitudinal factors and manual roughage provision, but also to the type of feeding gate. On farms with palisade gates cows were rated less fearful when being released from restraint compared to farms with diagonal gates. Sine 7 of 11 farms with palisade gates kept horned cows, this result might indirectly reflect associations with the herds´ horn status. However, this association might also indicate the measures´ vulnerability to confounding effects of the feeding gate design. Particularly in horned cows, palisade gates may ease leaving the feeding place. These results together with previous findings on limited repeatability of TTI (r_s_ = 0.33) and RB (r_s_ = 0.56) after a time interval of three weeks ([25]) suggest that TTI and RB are less appropriate measures for the on-farm assessment of the HAR.

Final models regarding AD (ADmedian, ADtouch and AD100) had relatively high explained variances (adj. R^2^ = 0.48 to 0.52). The associations to factors according to predictions support the measure´s general validity. The same is true for the final model regarding QBA that comprised the highest number of factors (four factors) and had the highest explained variance (adj. R^2^ = 0.70). Thus, beside the established AD measure, QBA appears to be a promising alternative measure, which should further be considered in future research.

### Influencing factors on the cows´ behaviour towards humans

The presented multivariable models showed that cows´ behaviour towards humans in the barn is related to attitudinal factors, but also to quantity and quality of human-animal contact, herd and management characteristics.

#### Stockpersons´ attitudes

Factors of stockpersons´ attitudes, which have been shown to be related to their behaviour towards animals and decisions concerning immediate handling in various previous investigations (e.g. [7,9]), were significant predictors for cows´ behavioural reactions in the major part of models. Stronger agreements on the importance of positive human-animal contacts (importancePOS) or stronger positive perceptions of voluntary human-animal contacts (contactVOL) were associated with herds showing less avoidance behaviour and reacting less fearful towards humans. This confirms results of Ivemeyer et al. [17] in Swiss dairy herds, where the extent of stockpersons´ agreement on the importance of contact with their animals correlated negatively with herd medians of AD and with percentages of cows that avoided an approaching human at ≥ 100 cm. Also in French dairy herds stockpersons´ attitudes were significantly associated with cows´ avoidance behaviour towards humans into the same direction [22].

The consistent associations of stockpersons´ attitudes with the cows´ behaviour support the argumentation that they form a basis for stockpersons´ behaviour and the quality of human-animal interactions.

#### Human-animal contact

Several proxies of more positive and frequent human-animal interactions were related to less fearful cows in different HAR-tests. For instance, a higher frequency of a range of voluntary human-animal interactions with dry cows was significantly related to a higher percentage of cows that allowed touching (ADtouch). Voluntary contact towards lactating cows, on the contrary, did not remain in any final model. Apparently, the care of dry cows stronger reflects additional efforts and emphasis on animal care. In line with this, manual provision of concentrates, a regular rewarding human-animal contact, was significantly associated with less fearful cows regarding QBAmedian and manual provision of roughage by tendency (p = 0.055) with less fearful cows regarding RBhigh. The stockpersons´ ability to identify individual cows is an additional measure that will depend on the duration and intensity of contact to the animals. In the present study, the identification of individual cows was associated with lower ADmedian and higher ADtouch.

Comparable patterns were reported in previous investigations: Waiblinger et al. [23] found intensity and continuity of contact (including e.g. frequency of brushing, identification of cows, and gentle handling) the strongest predictor for cows´ avoidance behaviour in the barn and at the feeding place. Ivemeyer et al. [17] found significant effects of the average contact time per day and cow and the stockpersons´ ability to identify individual cows on AD in the barn.

Against expectations, staff changes more than once a year, compared to no staff changes within the last years, were associated with less fearful cows regarding QBAmedian. In a previous investigation [23], on the contrary, the frequency of staff changes correlated positively with cows´ AD. Possibly, the association found within the present sample can be attributed to confounding farm effects: farms with more frequent staff changes were predominantly organic farm communities (8 of 12 farms), keeping smaller herds and regularly engaging trainees which was also related to higher contact times per cow (median = 8.7 on farms with frequent staff changes compared to median = 3.5 on farms with no staff changes) and to less cows per stockperson (median = 13.8 compared to median = 29.4).

#### Herd and management factors

With regard to herd and management factors, the percentage of dehorned cows (excluding genetically hornless cows) was significantly associated with higher ADmedian, with more fearful cows during tactile interaction (TTIhigh), and by tendency with higher AD100. This effect might not only reflect the animals´ negative experience with humans early in life, but also reflect joint aspects of specific farm types. Within the investigated sample, farms keeping horned herds had increased contact intensities, e.g. in terms of voluntary contacts to dry cows (mean = 57.4% compared to the total mean = 47.5%) and the percentage of farms providing roughage manually (73% compared to 47% of all farms). Farms with horned cows may need to undertake more efforts to successfully keep horned dairy cows in loose housing which includes maintenance of a good human-animal relationship [37].

Herd sizes were only associated with a higher percentage of cows that avoided the approaching human at ≥ 100 cm (AD100), which might be explained by a wider cow to stockperson ratio and accordingly reduced contact times between human and cow in larger herds. Within the investigated sample, herd size correlated significantly with the number of cows per stockperson (r_s_ = 0.65) and with contact time per cow (min/d) (r_s_ = -0.46) (S2 Table). This is in line with previous findings by Waiblinger and Menke [24] that the influence of herd size on the percentage of cows that allowed touching in the AD test is mediated by the intensity of stockpersons´ contact with cows. Herd size was significantly associated with the frequency of brushing, and the stockpersons´ ability to identify cows [24].

In the present study, selection for docility was a significant predictor for the proportion of cows that could be touched (ADtouch). This may partly reflect the genetic component of cows´ responsiveness towards humans. The estimated heritability of breeding traits referring to cattle behaviour in handling situations ranged from h^2^ = 0.7–0.53 (reviewed by [38,39]). However, at the same time, selection for docility may indicate a specific quality of relationship towards the animals, as mentioned for the other factors before. In a previous investigation, Ivemeyer [19] found farmers selecting their cows for docility performing by tendency (p = 0,062) more positive interactions during milking.

Furthermore, the milking system was related to QBAmedian: cows on farms with tandem milking parlour had lower QBAmedians, i.e. were less fearful, compared to farms with AMS. Although the low number of farms with tandem parlour (n = 4) within the investigated sample has to be considered, the differences between milking systems might be attributed to a calm milking routine in tandem parlours including shorter latencies when entering the parlour [40], and to a better visual contact between milker and cow as well as additional possibilities for tactile interactions, e.g. at the cows´ head region. However, again the choice of parlour type may also be associated with different types of farmers, who put differing emphasis on human-animal interactions. The same may be true for the difference between farms with tandem parlour and AMS, although it can be speculated that automatic milking may reduce direct human-cow interactions. However, in French dairy herds, on the contrary, according to univariable analyses a higher percentage of cows allowed touching in the AD test on farms with AMS compared to farms with milking parlour [22].

Although cross-sectional studies such as the present investigation cannot prove causality of an association, the results conform to a number of previous findings. They strongly suggest stockpersons´ attitudes to be a key factor for improving cows´ responsiveness towards humans, even though they affect the animals only indirectly via related management decisions or consequential behaviour [41]. Attitudes are relatively easy and quick to record on-farm or via postal questionnaire [8,42], while a standardised valid recording of stockperson behaviour during routine work and on a broad range of different farm types including farms with AMS has not been developed yet and is challenging regarding feasibility in epidemiological on-farm investigations.

## Conclusions

The present study identified patterns of associations between different animal-based HAR measures and human, herd, housing and management factors of different farm types including farms using AMS that confirm earlier findings: positive stockpersons´ attitudes towards the animals as well as frequent and positive human-animal contacts or conditions that promote such contacts are associated with a better HAR, i.e. with less fearful reactions of the cows towards humans. Highest explained variances were found for the outcome variables AD and QBA.

## Supporting information

S1 Code Sheet (XLSX)

S1 Table Univariable correlations between metric factors. (XLSX)

S2 Table. Cow behaviour at farm level (XLSX)

S3 Table Stockpersons´ attitudes at farm level. (XLSX)

S4 Table Farm factors. (XLSX)
